# Methotrexate Increases Skeletal Muscle GLUT4 Expression and Improves Metabolic Control in Experimental Diabetes

**DOI:** 10.1155/2012/132056

**Published:** 2012-06-13

**Authors:** Giuseppina T. Russo, Letteria Minutoli, Alessandra Bitto, Domenica Altavilla, Eugenio Alessi, Annalisa Giandalia, Elisabetta L. Romeo, Maria Francesca Stagno, Francesco Squadrito, Domenico Cucinotta, Jacob Selhub

**Affiliations:** ^1^Department of Internal Medicine, University of Messina, 98122 Messina, Italy; ^2^Section of Pharmacology, Department of Clinical and Experimental Medicine and Pharmacology, University of Messina, 98122 Messina, Italy; ^3^Vitamin Bioavailability Laboratory, Jean Mayer USDA Human Nutrition Research Center on Aging, Tufts University, Boston, MA 02111, USA

## Abstract

Long-term administration of 5-aminoimidazole-4-carboxamide ribonucleoside (AICAR) mimics the effects of endurance exercise by activating AMP kinase and by increasing skeletal muscle expression of GLUT4 glucose transporter. AICAR is an intermediate in the purine *de novo* synthesis, and its tissue concentrations can be increased, *in vivo*, by low doses of methotrexate (MTX) through the inhibition of the enzyme AICAR transformylase. We report here the first evidence that, in experimental type 2 diabetes, chronic treatment with low doses of MTX increases skeletal muscle GLUT4 expression and improves metabolic control. MTX (0.5 mg/kg body weight) or vehicle was administered intraperitoneally, once a week for 4 weeks, to genetically diabetic female C57BL/KsJ-*m* ^+^/^+^ *Lept* ^*db*^ mice (*db* ^+^/*db* ^+^) and their normoglycemic littermates (*db* ^+^/^+^ *m*). In the *db* ^+^/*db* ^+^ mice, MTX treatment was associated with a *∼*2-fold increase in skeletal muscle GLUT4 protein concentration and a >4-fold increase in GLUT4 mRNA expression (*P* < 0.01, all), as compared to vehicle-treated mice; no significant differences were noted in controls. MTX treatment was also associated with a significant reduction of glucose and insulin serum concentrations in diabetic mice (*P* < 0.001), and glucose levels only (*P* < 0.05) in controls. These data indicate a different route to increase skeletal muscle GLUT4 expression, through the potential inhibition of the enzyme AICAR transformylase.

## 1. Introduction

Skeletal muscle glucose uptake is the rate-limiting step of glucose utilization, and it is physiologically regulated by an insulin-dependent and an insulin-independent signaling pathways, both leading to the translocation of GLUT4 glucose transporter to the plasma membrane [1].

While insulin-stimulated glucose utilization is impaired in type 2 diabetes, physical exercise results in regular GLUT4 translocation and glucose uptake [2–4], mediated by the activation of 5′-AMP-activated kinase (AMPK), a cellular “fuel sensor” which detects ATP depletion induced by several conditions [3–9]. 

Several evidences indicate that the levels of GLUT4 expression in skeletal muscle are crucial for the regulation of total body glucose homeostasis [10–12]. Accordingly, the AMPK-induced increase of muscle GLUT4 content has become a potential pharmacological target to ameliorate glucose control, as also indicated by *in vitro* and *in vivo* studies with exogenous administration of different compounds, including the nucleoside 5-aminoimidazole-4-carboxamide ribonucleoside (AICAR) [13–19].

Notably, AICAR is also a naturally occurring molecule, an intermediate in the purine *de novo* synthesis, which is metabolized by AICAR transformylase, a folate-dependent enzyme which catalyzes the conversion of AICAR to formyl-AICAR, using 10-formyl tetrahydrofolate (THF) as donor of the formyl group.

Methotrexate (MTX), an anti-inflammatory and immunosuppressive drug commonly used in several chronic inflammatory disorders such as rheumatoid arthritis [20–22], is a non competitive inhibitor of AICAR transformylase [23]. The inhibition of this enzyme may lead to an upstream accumulation of AICAR, which in turns determines an increase of adenosine-5′-phosphate and adenosine levels, that are responsible for the anti-inflammatory and the potential atheroprotective effects of MTX [24–28].

Thus, it has been shown that a 4-week treatment with intermittent low doses of MTX, comparable to those currently used to treat chronic inflammatory disorders, was associated with a severalfold increase of AICAR concentration in splenocytes [26].

In the present study, we tested the hypothesis that the same weekly regimen with low doses of MTX [26] would increase skeletal muscle GLUT4 expression and improve glucose control in a mouse model of type 2 diabetes. These effects may be mediated by the MTX-related inhibition of AICAR transformylase, leading to an upstream accumulation of AICAR, which in turn may activate AMPK and its downstream pathways regulating GLUT4 expression. 

## 2. Materials and Methods 

### 2.1. Animals and Experimental Protocol

The research was reviewed and approved by the institutional animal care and use committee of the University of Messina.

Genetically diabetic female C57BL/KsJ-**m*^+^/^+^*Lept*^*db*^* mice (*db* ^+^/*db* ^+^) and their normal littermates (*db* ^+^/^+^ *m*) were obtained from The Jackson Laboratory (Bar Harbor, MEUSA). *db* ^+^/*db* ^+^ mice are a genetic model of type 2 diabetes that display many of the characteristics of the human disease (including hyperglycemia, insulin resistance, and obesity) and a marked decrease in skeletal muscle glucose utilization. The animals were 14 weeks old at the start of the experiments. They were obese, weighing 40–50 g, compared with their nondiabetic littermates, which weighed 25–32 g. During the experiments, the animals were housed one per cage, maintained under controlled environmental conditions (12 hour light/dark cycle, temperature approximately 23°C).

Animals were provided with water *ad libitum* and a low-folic acid diet (TD00434, Teklad Diets distributed by Harlan Laboratories, Italy). 

Both diabetic and control animals were divided into four subgroups (7 animals each). The first (diabetic) and second (control) subgroups were given weekly intraperitoneal (i.p.) injections (1 mL, using 1 cc syringe and 30 gauge needle) of MTX USP at the dose of 0.5 mg/kg body weight (MTX groups) for 4 weeks; the other two subgroups of diabetic and control mice were treated with pyrogen-free (USP) normal saline (0.9%) (vehicle groups) for 4 weeks [21]. There were no apparent adverse effects with either treatments that could be detected by visual inspection. 

At the end of each treatment period, mice were anesthetized with ketamine hydrochloride (110 mg/kg), sacrificed, and the hindlimb skeletal muscles were removed, snap-frozen, and stored at −80°C until analysis.

### 2.2. Glucose and Insulin Serum Levels' Measurements

Non-fasting blood samples for glucose and insulin assays were obtained from the retro-orbital plexus. Retro-orbital blood was drawn in the morning, twenty-four hours after the last MTX injection, promptly centrifuged, and serum was stored at −80°C until analysis.

 Serum glucose concentration was measured by a glucose-oxidase method (Biosystems S.A., Barcelona, Spain), and serum insulin concentration was determined using a mouse insulin ELISA kit (Linco Research, Inc., MO, USA). Insulin resistance was calculated by the homeostasis model assessment (HOMA_IR_) [29]. 

### 2.3. Skeletal Muscle GLUT4 mRNA Expression

GLUT4 mRNA content in hind limb skeletal muscle was measured according to the method reported by Buhl et al. [30]. Total RNA was isolated from skeletal muscle with OMNIzol reagents. cDNA was made using random primers as described by the manufacturer. Afterwards, PCR Master Mix containing specific primers and Taq polymerase was added. The specific primers were *GLUT-4 sense*: TTC TGG CTC TCA CAG TAC TC; *GLUT4 reverse*: CAT TGA TGC CTG AGA GCTGT; *β-actin sense: *TGG AAT CCT GTG GCA TCC ATG AAA C; *β-actin reverse: *TAA AAC GCA GCT CAG TAA CAG TCC G. The PCR products were stained with ethidium bromide, loaded on agarose gel for electrophoresis, and visualized at UV light.

### 2.4. Skeletal Muscle GLUT4 Protein Content 

Total**  **GLUT4 protein content in hind limb skeletal muscle extracts was measured by western blotting technique, using specific monoclonal antibodies. The primary antibody was a rabbit affinity purified polyclonal anti-glut-4 (catalog number CBL243 from Chemicon, Temecula, CA, USA).

#### 2.4.1. Protein Preparation

Muscles were removed and stored at −80°C until analysis. Tissues were weighed and homogenized in 10 mL of ice-cold buffer containing 100 mM HEPES pH 7.6, 150 mM NaCl, 5 mM EDTA, 5 mM MgCl_2_, 1% Triton X-100, and the following protease inhibitors 2 mM phenylmethylsulphonyl fluoride (PMSF), 5 mg/mL leupeptin, 1 mg/mL Pepstatin, 1 mg/mL aprotinin, and the following phosphatase inhibitor 100 mM sodium orthovanadate, 10 mM sodium fluoride, 10 mM sodium pyrophosphate. The homogenate was centrifuged at 10000 g for 20 min at 4°C, and the resulting supernatant was centrifuged at 9000 g for 20 min at 4°C.  The protein content of the final supernatant was determined by the Bradford protein assay (Bio-Rad) using BSA standards. 

#### 2.4.2. Determination of GLUT4 by Western Blot Analysis

Protein samples (40 *μ*g) were denatured in reducing buffer (62 mM Tris pH 6.8, 10% glycerol, 2% SDS, 5%  *β*-mercaptoethanol, 0.0035 bromophenol blue) and separated by electrophoresis on an SDS (12%) polyacrylamide gel. The separated proteins were transferred on to a nitrocellulose membrane using the transfer buffer (39 mM glycine, 48 mm Tris pH 8.3, 20% methanol) at 200 mA for 1 h. The membranes were blocked with 5% nonfat dry milk in TBS-0.1% Tween for 1 h at room temperature, washed three times for 10 min each in TBS-0.1% Tween, and incubated with a primary GLUT-4 antibody (Chemicon, Temecula, CA, USA) in TBS-0.1% Tween overnight at 4°C. 

After being washed three times for 10 min each in TBS-0.1% Tween, the membranes were incubated with a second antibody peroxidase-conjugated goat anti-rabbit immunoglobulin G (Pierce) for 1 h at room temperature. After three times washing for 10 min each in TBS-0.15%, the membranes were analyzed by the enhanced chemiluminescence system according to the manufacture's protocol (Amersham). 

The GLUT4 protein signal was quantified by scanning densitometry using a bioimage analysis system (Bio-Profil). The results from each experimental group were expressed as relative integrated intensity compared with control normal muscles measured with the same batch. 

### 2.5. Statistical Analysis

The results were expressed as mean ± SD. Data were analyzed by unpaired Student's *t*-test. When the number of groups analyzed was greater than two, analysis of variance was used. The level for statistical significance was set at *P* < 0.05.

## 3. Results

The effects of 4-week MTX or vehicle treatments on GLUT4 mRNA and protein expression were compared in diabetic (*db* ^+^/*db* ^+^) and control (*db* ^+^/^+^ *m*) mice.

**Figure 1:**
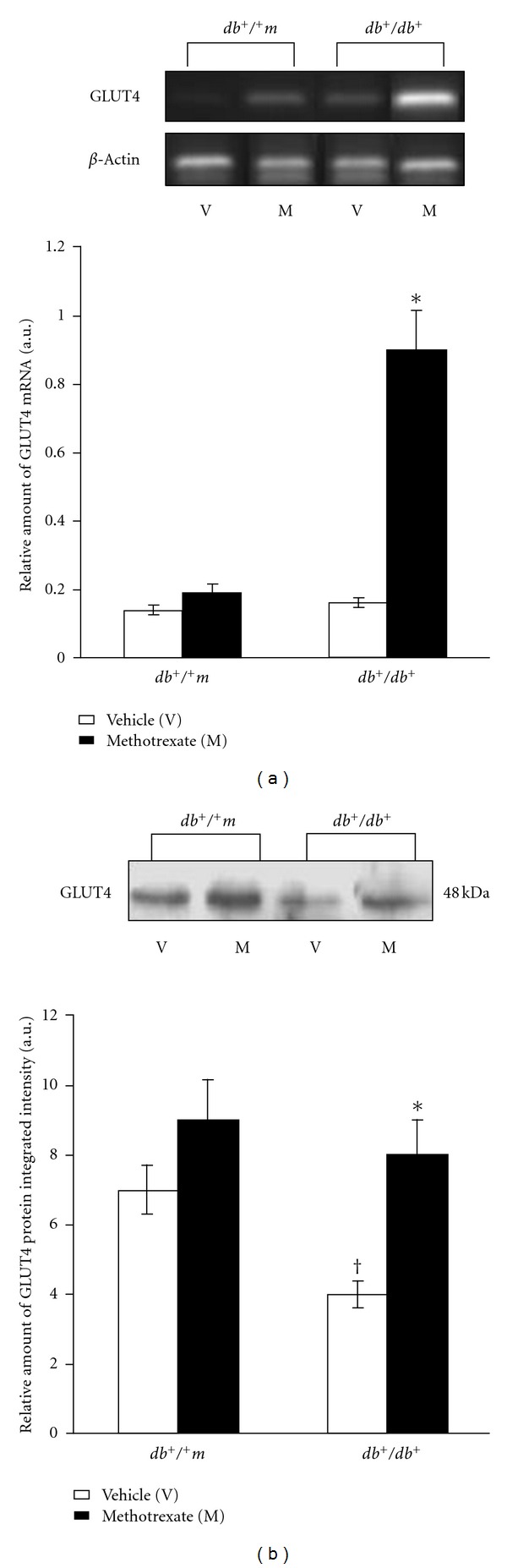
(a)  GLUT4 mRNA expression in muscle specimens collected from normoglycemic *db* ^+^/^+^ *m* mice and diabetic *db* ^+^/*db* ^+^ mice treated with either vehicle (white bars) or methotrexate (black bars). The upper panel shows representative gels highlighting GLUT4 mRNA expression. The lower panel shows quantitative data and represents the mean ± SD of seven experiments. *P < 0.01 versus *db* ^+^/*db* ^+^ plus vehicle. (b) Western blot analysis of the GLUT4 protein in muscle specimens from normoglycemic *db* ^+^/^+^ *m* mice and diabetic *db* ^+^/*db* ^+^ mice treated with either vehicle (white bars) or methotrexate (black bars). The upper panel shows representative autoradiography highlighting GLUT4 protein expression. The lower panel shows quantitative data and represents the mean ± SD of seven experiments. *P < 0.01 versus *db* ^+^/*db* ^+^ plus vehicle; ^†^ *P* < 0.05 versus *db* ^+^/^+^ *m* plus vehicle.

Total GLUT4 mRNA levels in *db* ^+^/^+^ *m* and *db* ^+^/*db* ^+^ mice chronically treated with MTX or vehicle are shown in Figure 1(a). Skeletal muscle total GLUT4 mRNA levels were comparable between diabetic and control mice treated with vehicle. In *db* ^+^/*db* ^+^ mice, MTX treatment was associated with a >4-fold increase in GLUT-4 mRNA content as compared to vehicle (*P* < 0.01), whereas in *db* ^+^/^+^ *m* mice the difference between MTX and vehicle groups was not significant.

As shown in Figure 1(b), total GLUT-4 protein content in skeletal muscle was significantly lower in vehicle-treated *db* ^+^/*db* ^+^  than in *db* ^+^/^+^ *m* mice (*P* < 0.05). In *db* ^+^/*db* ^+^  MTX-treated mice, there was a ~2-fold increase in total skeletal muscle GLUT-4 protein concentration as compared to those treated with vehicle (*P* < 0.01), whereas the difference between MTX- and vehicle-treated groups was not significant in controls (*db* ^+^/^+^ *m*). 

Effects of MTX or vehicle on metabolic control were then evaluated in both groups (Table 1). As compared to baseline values, *db* ^+^/*db* ^+^ animals treated with MTX showed a marked reduction of glucose and insulin serum levels (*P* < 0.001), whereas these differences were not statistically significant in the vehicle-treated group. 

Serum glucose and insulin concentrations did not significantly differ from baseline values after vehicle administration in controls (*db* ^+^/^+^ *m*) (Table 1). In this group, MTX treatment was associated with a lower, though still significant, decrease of serum glucose levels (*P* < 0.05), whereas insulin concentrations remained unchanged from baseline values.

Furthermore, MTX treatment was associated with a significant reduction of insulin resistance (HOMA_IR_) in both diabetic *db* ^+^/*db* ^+^ and control *db* ^+^/^+^ *m* mice (*P* < 0.001), whereas this decrement was not significant in the vehicle-treated groups (Table 1). 

In all the study groups, both daily food intake and body weight did not significantly change throughout the experiments (Table 1).

## 4. Discussion 

In the present study we tested the hypothesis that weekly low doses of MTX would increase skeletal muscle GLUT-4 expression and improve metabolic control in a mouse model of type 2 diabetes. 

This hypothesis was based on the fact that MTX is a noncompetitive inhibitor of AICAR transformylase, the folate-dependent enzyme that metabolizes AICAR to formyl-AICAR [23, 26]. The hypothesis predicts that blockage of this enzyme will lead to the upstream accumulation of AICAR [26], a well-known activator of AMPK and of its downstream pathways, which regulates insulin-independent GLUT4 expression and glucose metabolism [31, 32]. Accumulated AICAR is concerted to be converted in monophosphorylated nucleotide 5-aminoimidazole-4-carboxamide ribonucleotide (ZMP), which is able to activate AMPK, by mimicking the effects of AMP [13]. 

Short-term incubation of both cardiac and skeletal muscles with AICAR has been demonstrated to rapidly increase AMPK activity, to induce GLUT4 translocation on plasma membrane, and to stimulate glucose uptake [33–35]. Moreover, long-term activation of AMPK-dependent pathways, by endurance exercise or AICAR chronic administration, has been shown to increase GLUT4 expression, both at the protein and the mRNA levels [15–19].

There is a good evidence that the level of GLUT4 expression in skeletal muscle influences total body glucose homeostasis [10, 11]. The impairment of glucose uptake by the skeletal muscle GLUT4 is a primary defect in type 2 diabetes [1, 3, 4]. 

Total GLUT4 concentrations in skeletal muscle have been demonstrated to correlate with muscle glucose uptake capacity and whole body glucose disposal [12]. This finding is further supported by several observations. The selective disruption of GLUT4 transporter in mouse muscle results in a severe insulin resistance and glucose intolerance [11], whereas its overexpression ameliorates both glucose and lipid metabolism in transgenic mice [10]. Moreover, the expression of the human GLUT4 gene in the same strain of mice used in our experiments also resulted in improved basal glucose control, together with an increase in insulin sensitivity [36]. 

Our data suggest that GLUT4 expression can be induced by substances such as MTX that enhance the natural ability of cells to accumulate AICAR. 

In our experiments, the increase of GLUT4 expression in *db* ^+^/*db* ^+^ mice was associated with a marked decrease of both glucose and insulin concentrations, confirming that the level of expression of this transporter in skeletal muscle may be crucial for the regulation of total body glucose homeostasis and insulin resistance.

Similarly, the increase of GLUT4 expression induced by chronic AICAR administration was associated with a reduction of blood glucose levels [37] and an improvement of the other features of the metabolic syndrome [19]. These evidences suggest that the manipulation of GLUT4 expression by different means might be useful for ameliorating overall metabolic control in diabetes. Our results, demonstrating that MTX is able to increase GLUT4 expression and to improve glucose metabolism in diabetic animals, are in line with these observations.

Notably, beside the effects mediated by GLUT4 expression on metabolic control, AICAR accumulation may improve glucose levels also through a direct inhibition of gluconeogenesis. An in vitro study on isolated rat hepatocytes showed that AICAR was able to inhibit the production of glucose from several gluconeogenic precursors, in a dose-dependent manner, probably because of the inhibition of fructose-1,6-bisphosphatase by ZMP [38].

Nevertheless, since MTX is able to influence several other enzymes not thought to control AMPK activity [39], we cannot rule out the possibility that this drug might exert its effects on glucose metabolism also through mechanisms different from AICAR accumulation.

The anti-inflammatory properties of MTX may have helped to ameliorate the severity of diabetic pathology in our study, given the known involvement of inflammatory cytokines such as hsCRP, IL-6 in the development of insulin resistance, type 2 diabetes, and its long-term cardiovascular complications [40, 41]. MTX can modulate the expression of these cytokines, and recent studies suggest that it may also have a beneficial effect on cardiovascular mortality, which is not observed with other antirheumatic drugs [42].

Interestingly, Nf-kB pathway seems to be involved in the hypoglycaemic effects of anti-inflammatory drugs, and MTX has been shown to suppress NF-kB activation [43]. Furthermore, at the same doses used in our study, MTX improved diabetic nephropathy in another animal model of diabetes, through the inhibition of NF-kB pathway [44]. 

To date, the potential antidiabetic effects of NF-kB suppression induced by different anti-inflammatory drugs are currently being tested in several clinical trials [45–47]. Unfortunately, clinical data on the effects of MTX on glucose control are sparse and often influenced by the concomitant use of other drugs, such as corticosteroids. In a study on children with lymphoblastic leukemia (ALL), Halonen et al. reported that hypoglycaemia was among the side effects associated with high-dose MTX and 6-mercaptopurine (6MP) [48], but this effect was related to a reduced supply of neoglucogenic substrates [49]. Another small study in rheumatoid arthritis patients with type 2 diabetes reported that hydroxychloroquine determined a greater reduction of glucose control, as assessed by HbA_1c_ levels when compared to MTX, although these results were not adjusted for concomitant therapy with corticosteroids, that was more used in MTX patients [50].

Conversely, another intervention study in subjects with new onset type 1 diabetes reported that a combination therapy of cyclosporine and MTX induced a temporary remission of the disease with a decrease of the required insulin doses [51].

Although our study shows that MTX alone can improve glucose metabolism in animal model of type 2 diabetes and although MTX is widely prescribed as the main therapy for a number of chronic inflammatory disorders, its potential use for the chronic treatment of type 2 diabetes is not practical because of its toxicity and potentially harmful side effects. Nevertheless, our study suggests that targeting AICAR transformylase or using compounds that enhance intracellular AICAR production might be able to exert the same downstream effects of artificial AICAR administration on skeletal muscle GLUT4 expression and glucose homeostasis.

These findings open new perspectives, encouraging the search of other less harmful antifolates that, either by inhibiting AICAR transformylase or by increasing AICAR* in vivo *production, might have a potential application for the treatment of type 2 diabetes and other insulin-resistant conditions. 

## Figures and Tables

**Table 1:** Nonfasting glucose and insulin serum levels in genetically diabetic female C57BL/KsJ-*m* ^+^/^+^ *Lept* ^*db*^mice (*db* ^+^/*db* ^+^) and their normal littermates (*db* ^+^/^+^ *m*) at baseline and after 4 weeks of treatment with methotrexate (MTX) or vehicle.

		*db* ^+^/^+^ *m*		*db* ^+^/*db* ^+^	
		Vehicle	MTX	Vehicle	MTX
Body weight (gr)	Baseline	26 ± 4	27 ± 4	40 ± 7	42 ± 6
	After treatment	28 ± 5	26 ± 5	43 ± 5	39 ± 5
Glucose (mg/dL)	Baseline	185 ± 9	178 ± 10	543 ± 28	521 ± 19
	After treatment	177 ± 11	155 ± 8^∗^	499 ± 26	293 ± 15^#^
Insulin (pmol/L)	Baseline	68 ± 12	75 ± 7	165 ± 18	171 ± 14
	After treatment	64 ± 9	60 ± 6	159 ± 15	112 ± 11^#^
HOMA_IR_ (mmol/L∗*μ*UI/L)	Baseline	5.18 ± 0.92	5.49 ± 0.53	36.87 ± 3.9	36.66 ± 4.2
	After treatment	4.66 ± 0.64	3.83 ± 0.52^#^	32.65 ± 4.8	13.51 ± 2.2^#^

Data are means ± SD. Only significant *P* are presented. ^∗^ *P* < 0.05 versus baseline; ^#^ *P* < 0.001 versus baseline.
